# Effectiveness of Provider and Community Interventions to Improve Treatment of Uncomplicated Malaria in Nigeria: A Cluster Randomized Controlled Trial

**DOI:** 10.1371/journal.pone.0133832

**Published:** 2015-08-26

**Authors:** Obinna Onwujekwe, Lindsay Mangham-Jefferies, Bonnie Cundill, Neal Alexander, Julia Langham, Ogochukwu Ibe, Benjamin Uzochukwu, Virginia Wiseman

**Affiliations:** 1 Health Policy Research Group, Department of Pharmacology and Therapeutics, College of Medicine, University of Nigeria Enugu-Campus, Enugu, Nigeria; 2 Department of Global Health and Development, London School of Hygiene and Tropical Medicine, 15–17 Tavistock Place, London, United Kingdom; 3 Department of Infectious Disease Epidemiology, London School of Hygiene and Tropical Medicine, London, United Kingdom; 4 Faculty of Medicine and Health, University of Leeds, Leeds, United Kingdom; 5 Department of Medical Statistics, London School of Hygiene and Tropical Medicine, London, United Kingdom; 6 School of Public Health and Community Medicine, University of New South Wales, Sydney, 2052, Australia; The George Washington University School of Medicine and Health Sciences, UNITED STATES

## Abstract

The World Health Organization recommends that malaria be confirmed by parasitological diagnosis before treatment using Artemisinin-based Combination Therapy (ACT). Despite this, many health workers in malaria endemic countries continue to diagnose malaria based on symptoms alone. This study evaluates interventions to help bridge this gap between guidelines and provider practice. A stratified cluster-randomized trial in 42 communities in Enugu state compared 3 scenarios: Rapid Diagnostic Tests (RDTs) with basic instruction (control); RDTs with provider training (provider arm); and RDTs with provider training plus a school-based community intervention (provider-school arm). The primary outcome was the proportion of patients treated according to guidelines, a composite indicator requiring patients to be tested for malaria and given treatment consistent with the test result. The primary outcome was evaluated among 4946 (93%) of the 5311 patients invited to participate. A total of 40 communities (12 in control, 14 per intervention arm) were included in the analysis. There was no evidence of differences between the three arms in terms of our composite indicator (p = 0.36): stratified risk difference was 14% (95% CI -8.3%, 35.8%; p = 0.26) in the provider arm and 1% (95% CI -21.1%, 22.9%; p = 0.19) in the provider-school arm, compared with control. The level of testing was low across all arms (34% in control; 48% provider arm; 37% provider-school arm; p = 0.47). Presumptive treatment of uncomplicated malaria remains an ingrained behaviour that is difficult to change. With or without extensive supporting interventions, levels of testing in this study remained critically low. Governments and researchers must continue to explore alternative ways of encouraging providers to deliver appropriate treatment and avoid the misuse of valuable medicines.

**Trial Registration:**

ClinicalTrials.gov NCT01350752

## Introduction

Two key pillars of the World Health Organization’s (WHO) Guidelines for the Treatment of Malaria are parasitological confirmation of suspected malaria cases and the use of Artemisinin-based Combination Therapy (ACT) to treat confirmed cases of uncomplicated malaria. The move towards universal diagnostic testing is a critical step in the fight against malaria as it will allow for the targeted use of ACT in those who actually have malaria [1].

Rapid diagnostic tests for malaria (RDTs) have been widely promoted as a relatively cheap and effective way of encouraging diagnostic confirmation at all levels of the health system [2]. In practice, however, many factors undermine the effective uptake of the tests and adherence to current treatment guidelines [3]. These factors include a lack of effective provider training in administering RDTs in both the public and private sectors [2],[4], a distrust amongst both providers and patients in test results, especially negatives ones [5–12] and providers perceptions, including of patient expectations for certain treatments [3],[9],[13–14].

Prompt access to effective treatment for malaria is a key goal of the Nigeria Federal Ministry of Health [15] yet the provision and utilization of malaria treatment in south-eastern Nigeria remains poor [16]. Our formative research in 2009 revealed that only 13% of public facilities had microscopy available, and although ACT was introduced to Nigeria in 2005 only 55% of providers surveyed knew ACT was the recommended treatment. Moreover, while 79% of febrile patients received an antimalarial, only 23% of patients received an ACT and two-thirds of those were in the wrong dose. Approximately half of patients surveyed asked for a specific medicine, and in most cases this was not an ACT [9],[17].

If RDTs are to be an effective means of targeting the use of ACT in Nigeria then they must be supported by behaviour change interventions. This not only means changing established practices of providers but also changing the expectations of patients and their families [3]. This paper reports on a cluster-randomized trial to compare the effectiveness in the uptake of RDTs and adherence to malaria guidelines of: RDT supply plus i) a provider training intervention (provider arm) and ii) a provider training plus school-led community intervention (provider-school arm). The supply of RDTs with basic instruction defined the control arm and was selected to reflect expected practice by the Government of Nigeria in their planned roll-out of RDTs in 2012. The aim of the study was to evaluate the effectiveness of the interventions in the context of existing drug supply channels.

## Methods

The design of the trial and the interventions are described in detail elsewhere [18], a summary is outlined below. Both the protocol and CONSORT checklist of the trial are presented in S1 Protocol and S1 CONSORT Checklist.

### Study setting and population

The effects of the interventions were evaluated using a three-arm stratified, cluster randomized trial in 42 communities within urban Enugu and rural Udi Local Government Areas (LGAs). Since the school-based intervention was delivered at the community level, an eligible cluster was defined as a geographical community containing at least one facility and one school (primary or secondary), with LGA as the stratum.

Within a cluster, all consenting facilities and schools were eligible for inclusion in the study. Outcomes were assessed at patient, provider, facility and household level. All patients (or caregivers) attending participating facilities were approached on exit and screened for eligibility. Patients were eligible if they (or their caregiver) reported that the patient was: suffering from a fever or had a history of fever in this illness episode, and were present at the facility; not pregnant; more than 6 months old; and did not have signs or symptoms of severe malaria. At selected facilities all providers involved in the diagnosis and treatment of suspected cases of malaria were eligible. Households were eligible if any member reported having had a fever in the previous two weeks. The nature and purpose of the trial was explained to all participants and written informed consent obtained.

Ethical approval was obtained from the University of Nigeria (UNTH/CSA 329/Vol 6) and the London School of Hygiene and Tropical Medicine (No.5885). Written consent was obtained from primary caregivers on behalf of the minors/children enrolled in the study. This consent procedure was approved by both ethics committees. The trial is registered with clinicaltrials.gov NCT01350752.

### Selection of clusters, randomisation and masking

Within a stratum, communities were randomly selected from those eligible with probability proportional to size. Randomisation to the study arms was conducted through a process of restricted randomisation using a program written in R statistical software version 2.13.0 (R Foundation for Statistical Computing, Vienna, Austria) by the study statistician who had no involvement in the delivery or evaluation of the intervention. Within each community, schools were selected purposively, while facilities were selected at random with probability proportional to the community size in terms of the total number of facilities within the community. Selection and randomization was performed after community heads, schools and facilities had been informed of the study and provided written consent. Patients (or caregivers) and fieldworkers administering the surveys were masked to the assignment of study arm. It was not possible to blind participants or those responsible for implementing the interventions and supervising data collection.

### Interventions

An initial delivery of between 25–75 RDTs per month (SD Bioline Malaria Ag Pf/Pan) was made without charge to all participating facilities (with the exact amount depending on the average number of febrile patients that a facility expected during one month). Public facilities were asked not to charge patients, while private facilities were advised that a maximum of 100 Naira (0.6USD) was the recommended price per test. Facilities could request additional RDTs when they ran out of stock. Also, the providers had the option of prescribing the ACTs (as they were taught) if they ran out of stock on ACTs. The availability of ACT was not controlled and facilities were expected to receive their supply through their usual channels. Providers were however trained in prescribing ACT if they did experience stock-outs. The study team monitored the availability of ACTs during supervisory visits to the facilities and at the time of the provider survey. Our formative research showed that around 80% of facilities had ACTs in stock at the time of the survey [9].

The State Malaria Control Programme (SMCP) and the Association of Community Pharmacists and Association of Patent Medicine Dealers (PMDs) were involved in the design of the interventions. All training materials are available at http://www.actconsortium.org/.

#### Demonstration on how to use RDTs

Providers in the control arm were invited to a demonstration and practical on how to safely use RDTs and supplied with written instructions on their use. Depending on the size of a facility, 1 to 2 providers involved in the diagnosis and treatment of suspected cases of malaria were invited to the demonstration.

#### Provider intervention

Between 1 and 2 providers working in facilities assigned to the intervention arms were invited to a two-day training workshop and received support visits. The training was designed to be suitable for implementation on a large scale and covered the following topics: causes and symptoms of malaria; demonstration on how to use an RDT; the updated guidelines for malaria diagnosis and treatment; appropriate treatment when the malaria test is positive and negative; and communications skills. The topic on appropriate treatment explained that ACT should be given when a malaria test is positive, covered the different types of ACT and the dosage regimens by age group, explained that the WHO has recommended the use of RDT and advises that no antimalarial is needed when the test result is negative, addressed health worker prejudices of malaria tests such as mistrust of negative test results, and advised on other causes of febrile illness that could be investigated if the malaria test is negative. In the session on communication skills providers learnt how to discuss different treatment options with patients, especially when the test result is negative.

The training used a combination of seminars and facilitated small-group work, such as a treatment algorithm game, problem-solving exercises, self-developed participatory drama and role-playing. Providers attending the training received copies of training materials and job aids. The training was given by representatives from the SMCP and the research team. Facilities also received a support visit every month during the implementation and evaluation phases of the study to offer guidance to providers experiencing difficulties.

#### School-based community intervention

Communities randomised to the provider-school arm received the provider intervention and a school-based intervention which initially involved training two teachers per school on peer health education and how to hold a community event to raise awareness about malaria. Peer health education has been shown to influence the knowledge, attitudes, and practice of school children and their families as well as the wider community [19–22] and has been used recently by the Government in Enugu State to support community awareness and participation in onchocerciasis control activities [23–25]. The school teachers, with support from the research team, were then expected to train 12 school children as peer health educators (PHEs) and work with them to implement various activities to promote the use of RDTs and explain that ACT is the recommended treatment for uncomplicated malaria. The activities included dramas, songs, card games, and health talks and were undertaken during morning assembly, Parent-Teacher Association meetings, and at prize-giving days. In addition, teachers and PHEs were offered support to hold malaria events in which parents, guardians, and other community members could participate in the same types of activities. Posters, leaflets, T-shirts, and baseball caps promoting the school-based intervention were available for distribution at all events. A detailed description of each type of school-based activity can be found in S1 Protocol.

### Outcomes

#### Process evaluation

Data were collected on the process of implementing the interventions including: records of RDTs supplied; details of all participants attending the provider training; a structured pre-post training test to assess providers’ understanding of training material; an evaluation of participant satisfaction with the training; and a report on the roll-out of each workshop by those who facilitated the training. For the schools, the number of PHEs appointed, the number of malaria events held and attendance at these events were also recorded.

#### Impact evaluation

The primary outcome was the proportion of patients attending facilities that reported a fever or suspected malaria and received treatment according to the malaria guidelines. This is a composite measure where: 1) febrile patients should be tested for malaria, using either microscopy or RDT; 2) patients should be prescribed or receive ACT if they have a positive malaria test result; and 3) patients should not be prescribed or receive an antimalarial if they have a negative malaria test result. The primary outcome was assessed through an interviewer-administered patient exit survey which commenced 3 months after the intervention had been implemented. The exit survey was supplemented by a malaria test register completed by providers at each facility, since patients may not know if they were tested for malaria or the result of the malaria test. At each facility the research team collected the registers at least once a week and also independently conducted RDT tests in a sub-sample of 5% of patients to determine the accuracy of the reporting of the test results by the provider.

On the basis of our formative research [9] we assumed that the proportion of febrile patients receiving treatment according to malaria guidelines would be 10% in the control arm with a coefficient of variation between clusters within stratum (*k*) of 0.35 and a harmonic mean of 50 febrile patients per cluster. With 14 clusters per arm this would give 80% power to detect at least a (absolute) 15% incremental increase between each of the intervention arms and the control, and between the two intervention arms, at the 5% significance level.

**Fig 1:**
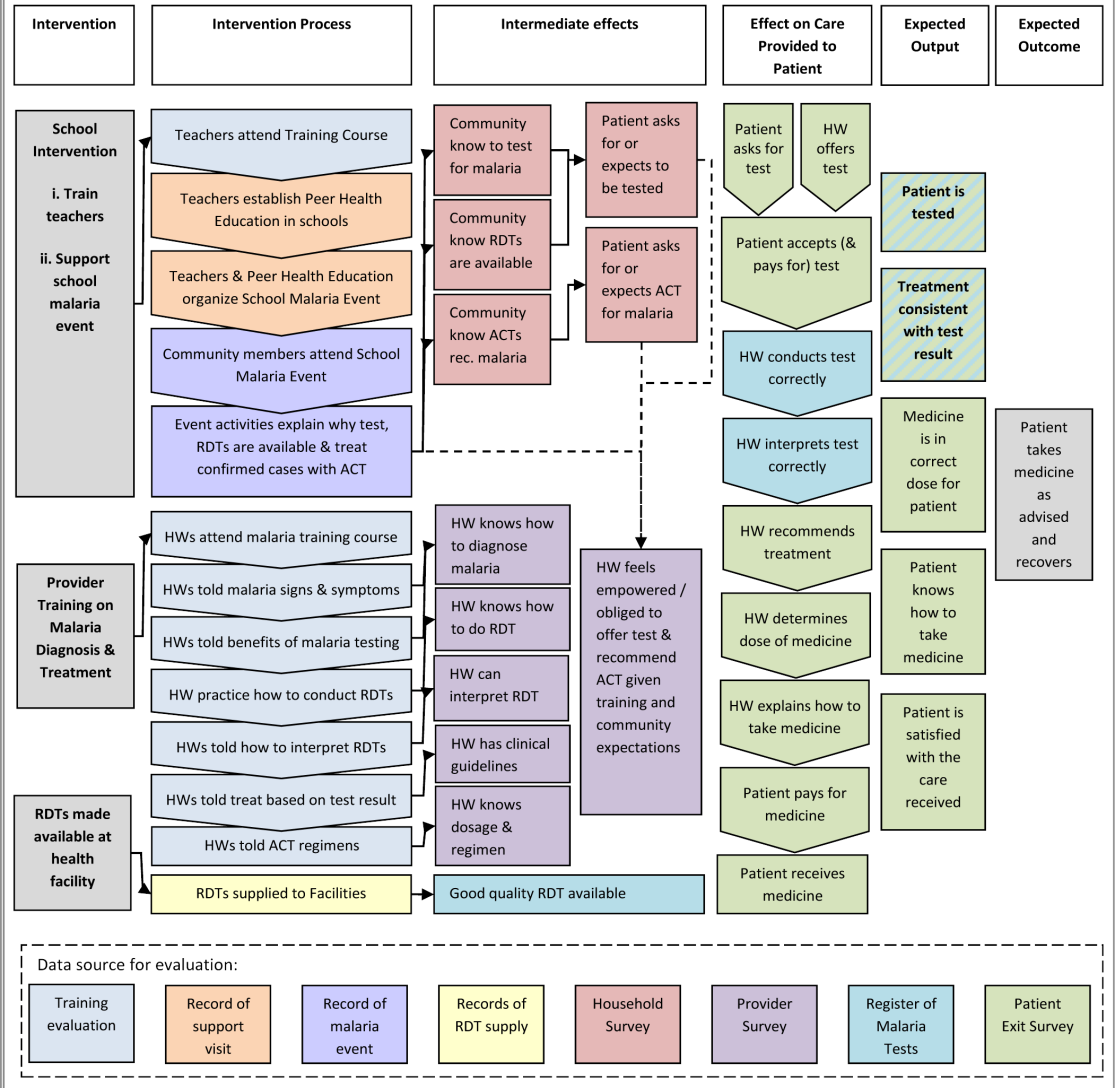
Logical framework.

A provider survey was conducted to measure changes in secondary outcomes related to the knowledge and ability to test and appropriately treat patients with suspected malaria. The provider survey was conducted after the patient exit survey to ensure the content did not influence treatment received by patients. A household survey was also completed within 3 months after intervention implementation, by one individual per household, to collect data on secondary outcomes associated with community knowledge of malaria diagnosis and treatment and to provide insight into the reach and impact of the school-based activities. The hypothesised effect of the interventions on providers and patients is outlined in the logical framework (Fig 1). Further details on the secondary outcomes and precision estimates for the provider and household survey are reported in the study protocol S1 Protocol.

#### Costs

The financial costs incurred to develop and implement the interventions were obtained from project financial accounts and implementation records. Data was also collected on the cost of diagnosing and treating cases of suspected malaria from health facility records and the provider and patient exit surveys.

### Statistical analysis

Data were entered and verified using Microsoft Access 2007and analysed using STATA version 11.0 (Stata corporation, College Station, TX, USA). All analysis was by intention to treat.

For the primary outcome, a point estimate was calculated for each cluster. These cluster-level summaries were analysed using two-way analysis of variance by stratum and study arm, to provide risk differences (RD) and 95% confidence intervals (CI) comparing each intervention arm relative to the control, and the intervention arms with each other. The F-test was used to assess the null hypothesis of no differences overall between study arms. Although the proportions were skewed, we did not log-transform cluster summaries because of zero events in some clusters and instead relied on the robustness of Gaussian procedures [26]. A weighted analysis [27] was not pursued because the between-cluster variation was high enough for these weights to be similar across clusters (range of weights 1.72–1.86).

Adjustment for covariates was made by a two-stage method similar to that proposed by Bennett et al [28]. The probability of appropriate treatment was fitted from a logistic regression model on individual-level data, including terms for stratum, the covariates of interest and arm. Expected numbers were computed, without the intervention effects, and compared with the observed values for each cluster. The methods for estimating the RDs and 95% CIs were calculated as before, but on the observed minus expected numbers.

Secondary outcomes were analysed as for the primary outcome without adjustment for covariates. Agreement between the different sources of data on whether or not a patient had a malaria test and the result of the malaria test was assessed using the kappa statistic.

## Results

**Fig 2:**
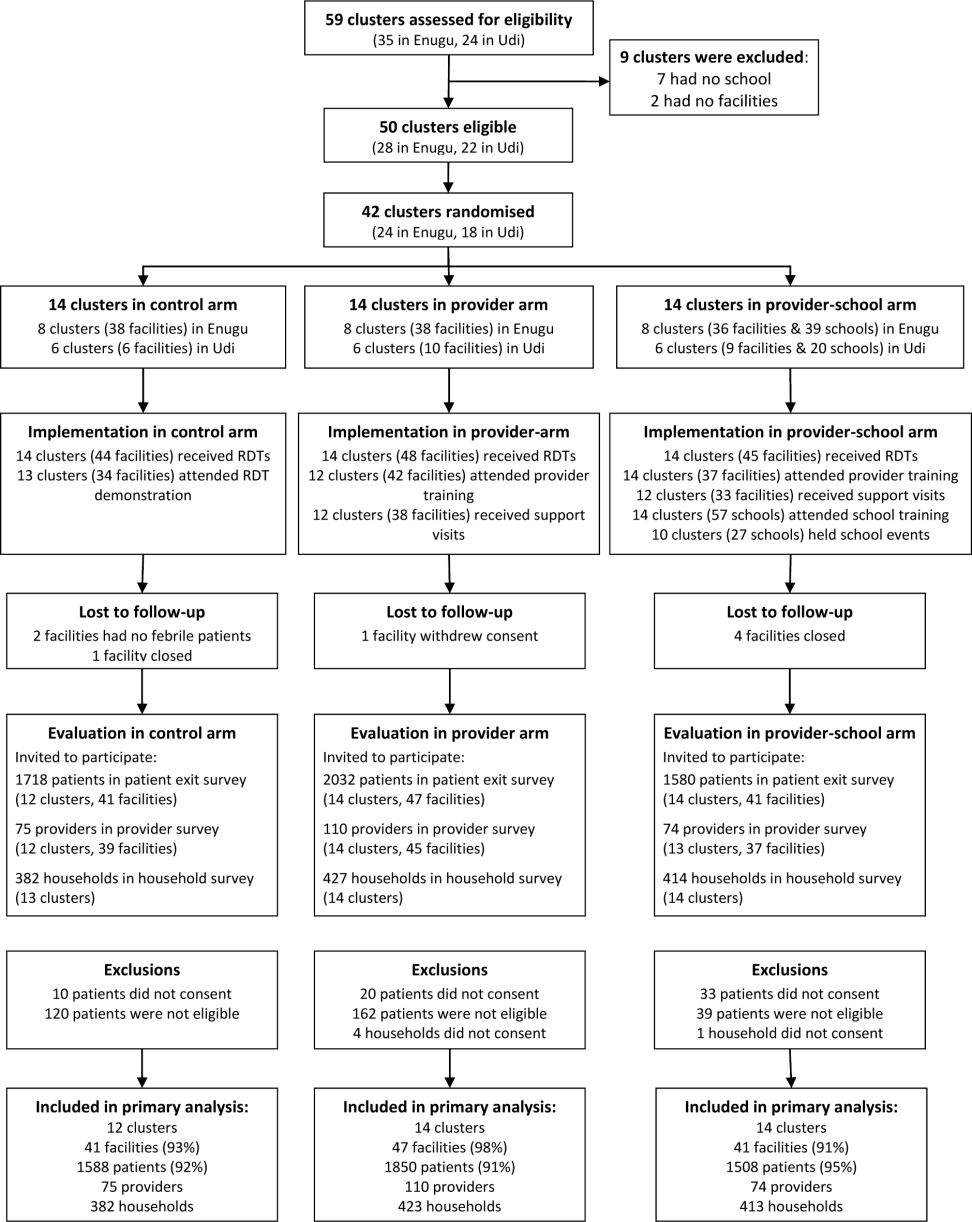
Trial profile.

The trial profile is provided in Fig 2. The study took place between 6^th^ June and 19^th^ December 2011. A total of 42 clusters (14 per arm) were randomised and 40 (12 in control, 14 per intervention arm) were included in the analysis. Two clusters in the control arm were not included in the analysis because no patients visited these health facilities. The primary outcome was evaluated among 4946 (93%) of the 5311 patients invited to participate in the study.

### Process evaluation

Of the 137 facilities that were invited and agreed to participate in the trial, 113 (82%; 125 providers) were represented at the RDT demonstration or provider training. Reasons for non-attendance included being too busy, short-staffed, travelling, bereavement, and sickness or death. The training workshops were successfully delivered, with 112/125 (90%) of participants strongly agreeing that they were satisfied with the delivery of the course by the trainers and the relevance of the material to their work. Participants’ knowledge of topics covered in the material increased from 59% to 82% (as measured by the pre-post training test). In the intervention arms 71/79 (90%) facilities received support visits, due to closure of some facilities on the visit days.

A total of 109 teachers from 59 schools were initially trained on peer health education and holding community events. Teachers from 57 schools subsequently trained 510 children as PHEs. Twenty-seven schools held a malaria event with considerable variation in terms of the range of activities undertaken, attendance (80 to 400 community members) and duration (1 to 4 hours). The evaluation of pre and post tests for training of school teachers showed an increase in knowledge from 12% to 50%.

Three facilities in each of the provider (6%) and control (7%) arm reported stock-outs of RDTs in the 4 weeks prior to the provider survey compared to 1 facility (2%) in the provider-school arm. All facilities procured ACT through the usual channels with a third of facilities in the provider (32%) and provider-school (33%) arms reporting stock-outs in the 4 weeks before the provider survey compared with 16% in control. Stock-outs of ACT mainly occurred in public facilities (86%) compared with pharmacies (5%) and drug stores (23%) due to an unexpected break in supply from the Central Medical Stores to public facilities.

### Characteristics of the study population

The stratification and restricted randomisation were shown to have provided study arms that were generally similar in their characteristics with a few exceptions (Table 1). Patients were more likely to attend pharmacies in the provider-school arm, and drug stores in the other arms. Those in the control arm appear to be of higher socio-economic status and of the highest (tertiary) education.

**Table 1:** Characteristics of clusters and patients, by study arm.

Characteristics	Control	Provider arm	Provider-school arm
**CLUSTER LEVEL**	**N**_**c**_ **= 12**	**N**_**c**_ **= 14**	**N**_**c**_ **= 14**
**Number per cluster (median, range)**			
Facilities	1 (1–16)	1 (1–15)	1 (1–10)
Patients	41 (34–593)	43 (15–596)	40 (11–393)
Schools	n/a	n/a	3.5 (1–13)
**Stratum**			
Enugu	7 (58%)	8 (57%)	8 (57%)
Udi	5 (42%)	6 (43%)	6 (43%)
**PATIENT LEVEL**	**N**_**p**_ **= 1588** ^**†**^	**N**_**p**_ **= 1850**	**N**_**p**_ **= 1508**
**Number of patients (median, range)**			
Per cluster	41 (34–593)	43 (15–596)	40 (11–393)
Per facility	40 (15–46)	40 (15–51)	40 (6–60)
**Stratum**			
Enugu	1386 (87%)	1473 (80%)	1270 (84%)
Udi	202 (13%)	377 (20%)	238 (16%)
**Facility type**			
Public	236 (15%)	340 (18%)	216 (14%)
Pharmacy	534 (34%)	600 (32%)	680 (45%)
PMD	818 (52%)	910 (49%)	612 (41%)
**Gender**			
Male	807 (51%)	927 (50%)	801 (53%)
Female	770 (49%)	913 (50%)	698 (47%)
**Age**			
<5 years	142 (9%)	166 (9%)	166 (11%)
5–19 years	312 (20%)	292 (16%)	214 (14%)
20–40 years	869 (55%)	910 (49%)	860 (57%)
≥40 years	265 (17%)	482 (26%)	268 (18%)
**Education of respondent** ^**‡**^			
None	73 (5%)	141 (8%)	102 (7%)
Primary	169 (11%)	270 (15%)	170 (11%)
Secondary	617 (39%)	756 (41%)	700 (47%)
Tertiary	711 (45%)	664 (36%)	520 (35%)
**Wealth index** ^**#**^			
Poorest	361 (25%)	612 (30%)	535 (42%)
Less poor	426 (30%)	625 (35%)	453 (35%)
Least poor	647 (45%)	55 (31%)	300 (23%)
**Days of illness** *			
Median (range)	2 (0–31)	2 (0–42)	2 (0–90)
**Seeking treatment for first time** *			
No	323 (21%)	388 (21%)	238 (16%)
Yes	1242 (79%)	1457 (79%)	1262 (84%)

TABLE NOTES

Numbers and percentages are presented unless stated otherwise.

^†^ N_p_ represents the number of patients who participated in the patient exit questionnaire (PEQ).

‡ Education level of respondent not known for 18 in control arm, 19 in provider arm and 16 in provider-school arm

^#^ Generated through principle component analysis and based on ownership of household possessions (e.g. electricity, radio, mobile phone, generator, bicycle, and car), access to utilities (toilet type and source of drinking water), and housing characteristics (floor type, fuel, persons per sleeping room) in line with DHS Wealth Index [29] and Vyas et al [30] use of PCA for SES.

* Refers to this illness episode. For those who had previously sought treatment for this illness episode 622 (74%) had sought treatment once before, while 138 (16%) had sought treatment twice before and 10% more than three times.

### Impact on treatment according to guidelines

Although clusters in the provider arm tended to have higher proportions of people treated according to guidelines (36% in provider, 23% control, 24% provider-school) there was no evidence of a statistically significant difference between the arms (p = 0.36) (Table 2). After adjustment for stratification and covariates of interest the proportion was on average 8% higher than control in the provider arm (95% CI -4.4%, 21.3%; p = 0.15) and 7% higher than control in provider-school arm (95% CI -5.7%, 20.0%; p = 0.17). There was also no evidence of a difference between the two intervention arms; adjusted RD was -12.9% (95% CI -34.0%, 8.3%) (Table 2 –Notes).

**Table 2:** Effects of the interventions on the primary outcome compared with control, by stratum.

Outcome	Study arm and stratum	Clusters	Individual-level prevalence	Cluster-level prevalence	Crude risk difference	Stratified risk difference^†^	F test^†^
		n	n/N (%)	Mean (SD)	RD (95% CI)	RD (95% CI)	p-value
**Febrile patients tested for malaria**	**Control**	**12**	**432/1536 (28%)**	**33.9% (29)**	**0**	**0**	**0.47**
	Enugu	7	339/1336 (25%)	24.8% (19)			
	Udi	5	93/200 (47%)	46.7% (38)			
	**Provider**	**14**	**416/1832 (23%)**	**48.3% (33)**	**14.4 (-11.2, 40.0)**	**14.2 (-11.0, 39.4)**	
	Enugu	8	287/1463 (20%)	50.0% (39)			
	Udi	6	129/369 (35%)	46.0% (28)			
	**Provider-school**	**14**	**231/1496 (15%)**	**36.5% (33)**	**2.5 (-23.0, 28.1)**	**2.4 (-22.8, 27.6)**	
	Enugu	8	133/1266 (11%)	24.8% (22)			
	Udi	6	98/230 (43%)	52.0% (41)			
**Test positive patients receiving ACT**	**Control**	**12**	**238/320 (74%)**	**64.2% (28)**	**0**	**0**	**0.76**
	Enugu	7	200/265 (75%)	71.2% (25)			
	Udi	5	38/55 (69%)	54.3% (32)			
	**Provider**	**14**	**95/142 (67%)**	**56.1% (31)**	**-8.1 (-33.3, 17.0)**	**-6.9 (-29.6, 15.8)**	
	Enugu	8	6/87 (79%)	73.8% (19)			
	Udi	6	26/55 (47%)	35.3% (31)			
	**Provider-school**	**12**	**59/98 (60%)**	**56.4% (33)**	**-7.8 (-33.5, 17.9)**	**-7.8 (-30.9, 15.4)**	
	Enugu	7	46/68 (68%)	67.5% (26)			
	Udi	5	13/30 (43%)	41.0% (38)			
**Test negative patients receiving an antimalarial**	**Control**	**11**	**51/112 (46%)**	**55.6% (38)**	**0**	**0**	**0.28**
	Enugu	6	34/74 (46%)	60.8% (40)			
	Udi	5	17/38 (45%)	49.4% (40)			
	**Provider**	**14**	**94/274 (34%)**	**30.9% (33)**	**-24.7 (-55.5, 6.2)**	**-24.9 (-56.0, 6.2)**	
	Enugu	8	70/200 (35%)	24.3% (28)			
	Udi	6	24/74 (32%)	39.7% (38)			
	**Provider-school**	**13**	**61/133 (46%)**	**43.7% (42)**	**-12.0 (-43.3, 19.4)**	**-12.5 (-44.2, 19.1)**	
	Enugu	8	48/65 (74%)	56.1% (48)			
	Udi	5	13/68 (19%)	23.7% (22)			
**Treatment according to malaria guidelines** ^‡^	**Control**	**12**	**299/1536 (20%)**	**22.6% (23)**	**0**	**0**	**0.36**
	Enugu	7	240/1336 (18%)	17.6% (15)			
	Udi	5	59/200 (30%)	29.6% (31)			
	**Provider** ^§^	**14**	**275/1832 (15%)**	**36.4% (32)**	**13.8% (-8.0, 35.7)**	**13.8% (-8.3, 35.8)**	
	Enugu	8	199/1463 (14%)	43.5% (38)			
	Udi	6	76/369 (21%)	27.0% (21)			
	**Provider-school** ^§^	**14**	**131/1496 (9%)**	**23.5% (26)**	**1.0% (-20.9, 22.9)**	**0.9% (-21.1, 22.9)**	
	Enugu	8	63/1266 (5%)	13.0% (14)			
	Udi	6	68/230 (30%)	37.6% (32)			

TABLE NOTES

**Fig 3:**
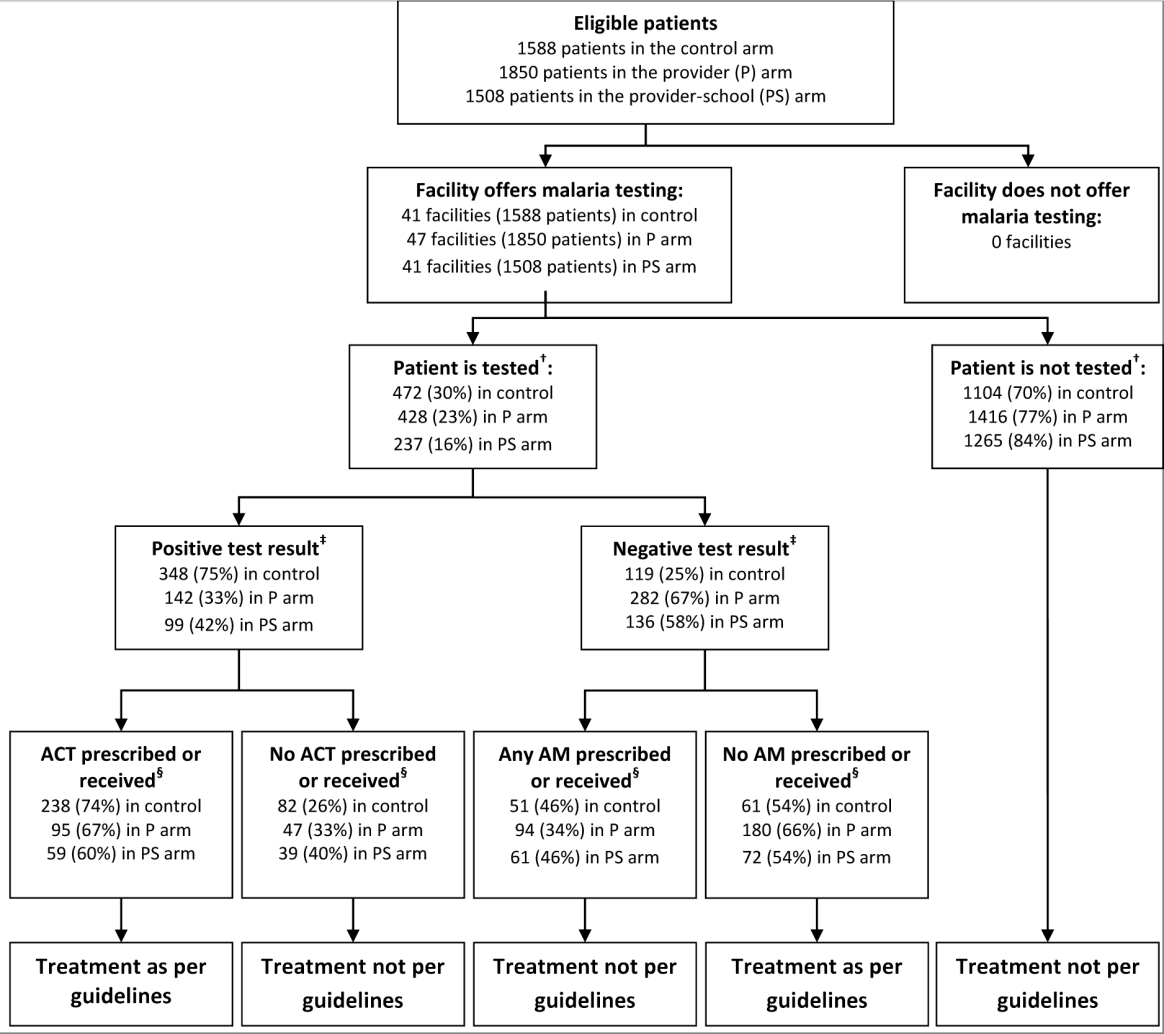
Flow chart showing definition of primary outcome.

ACT = Artemisinin-based Combination Therapy. Only those with complete data for all components of the primary outcome are included. Hence, for example, 1,079 people are shown as having been tested, 58 fewer than the 1,137 in Fig 3, due to the 11+29+18 missing values noted there.

^**†**^Stratified analysis of cluster-level summary measures, F test of the null hypothesis of no differences between the three treatment arms, therefore 2 numerator degrees of freedom.

^‡^The between-cluster coefficient of variation was 0.26 in the Provider arm, and 0.19 in the Provider-school arm. A further comparison between arms adjusted for: facility type, stock-out of ACTs in past 4 weeks, age and sex of patient, tertile of socio-economic status from principal component analysis, whether the patient had previously sought treatment, and whether they asked for a blood test. Missing values in these variables forced the omission of 577 people. Compared to control, this yielded a benefit of Provider of 8.4% (95% CI: -4.4 to 21.3%) and of Provider-school of 7.1% (95% CI: -5.7 to 20%).

^§^The stratified analysis of Provider-school arm versus Provider arm showed a difference of -12.9% (95% CI -34.0 to 8.3%).

Less than half of febrile patients attending the facilities were tested for malaria and of those tested, the proportion of patients who tested positive and received (or were prescribed) an ACT was lower in the intervention arms, but these differences were not statistically significant (Table 2; Fig 3). There was a decrease in the proportion of test negative patients who received (or were prescribed) an antimalarial in the intervention arms but again the differences were not statistically significant (Table 2; Fig 3). A larger proportion of patients in public facilities were tested compared to the private sector but there was no evidence of differences in testing or treatment according to results between arms in either sector (Table 3).

**Table 3:** Effects of the interventions on the primary outcome compared with control, by facility type.

Outcome	Study arm	Clusters^†^		Individual-level prevalence		Cluster-level prevalence		Stratified risk difference^‡^	
		Public	Private	Public	Private	Public	Private	Public	Private
		n	n	n/N (%)	n/N (%)	Mean (SD)	Mean (SD)	RD(95% CI)	RD(95% CI)
**Febrile patients tested for malaria**	Control	6	6	118/229 (51.5%)	314/1307 (24.0%)	50.0% (32)	17.9% (15)	0	0
	Provider	10	7	230/325 (70.8%)	186/1507 (12.3%)	69.2% (21)	16.9% (12)	22.7%(-5.6, 51.0)	0.5%(-15.2, 16.2)
	Provider-school	7	7	122/213 (57.3%)	109/1283 (8.5%)	61.8% (28)	11.1% (12)	13.1%(-16.7, 42.9)	-6.1%(-21.5, 9.3)
**Test positive patients receiving ACT**	Control	6	6	45/66 (68%)	193/254 (76%)	61.9% (34)	66.5% (23)	0	0
	Provider	10	7	40/73 (55%)	55/69 (80%)	57.3% (36)	51.3% (35)	-8.3%(-46.7, 30.0)	3.2%(-33.7, 40.2)
	Provider-school	7	7	16/37 (43%)	43/61 (70%)	48.6% (32)	64.3% (36)	-13.4%(-55.2, 28.5)	-2.2%(-34.6, 30.3)
**Test negative patients receiving an antimalarial**	Control	6	6	18/52 (35%)	33/60 (55%)	41.1% (41)	73.0% (29)	0	0
	Provider	10	7	27/157 (17%)	67/117 (57%)	24.7% (32)	33.0% (33)	-14.0%(-54.7, 26.7)	-37.6%(-83.3, 8.3)
	Provider-school	7	7	21/85 (25%)	40/48 (83%)	26.5% (38)	63.7% (41)	-13.8%(-56.7, 29.1)	-9.6%(-56.0, 36.7)
**Treatment according to malaria guidelines**	Control	6	6	79/229 (34%)	220/1307 (17%)	33.3% (27)	11.8% (11)	0	0
	Provider	10	7	170/325 (52%)	105/1507 (7%)	52.3% (28)	10.0% (8)	20.1%(-10.5, 50.8)	-0.7%(-12.3, 11.0)
	Provider-school	7	7	80/213 (38%)	51/1283 (4%)	40.7% (26)	6.4% (9)	7.8%(-24.6, 40.1)	-4.9%(-16.3, 6.5)

TABLE NOTES

^**†**^A cluster contributes to the analysis of public facilities if it has at least one such facility, and similarly for private. Clusters with at least one of each type contribute to both analyses.

^‡^The stratified risk differences were calculated as before, but separately for public and private facilities.

Agreement between RDT reporting in registers and the patient exit survey was very high for RDT testing (98% agreement; κ = 0.94) and test results (97% agreement; κ = 0.95). The observed agreement between the readings from the independent testing by the study team and the malaria registers was also excellent (96%; κ = 0.93).

### Impact on provider and community knowledge

There was no evidence of a difference by arm in the proportion of providers who knew the treatment guidelines or reported they would not give an antimalarial for test negative cases (Table 4). Almost all providers knew that febrile patients should be tested for malaria although a markedly higher proportion of providers in the intervention arms reported that they would give an ACT if a malaria test was positive (83% in control, 98% in provider arm and 99% in provider-school arm). Community knowledge was marginally higher among households in the intervention arms but differences were not statistically significant.

**Table 4:** Impact on provider and community knowledge of malaria diagnosis and treatment, by study arm.

	Arm	Clusters	Individual-level prevalence*N (%)	Cluster-level prevalenceMean (SD)	Stratified RD (95% CI)	F-test (p-value)
**PROVIDER KNOWLEDGE**						
The treatment guidelines^†^	Control	12	40/74 (54%)	67% (31%)	0	0.72
	Provider	14	57/109 (52%)	62% (33%)	-5.2% (-33.4, 23.1)	
	Provider-school	13	52/74 (70%)	57% (44%)	-11.5% (-40.2, 17.3)	
Febrile patients should be tested for malaria	Control	12	71/75 (95%)	95% (8%)	0	0.70
	Provider	14	105/109 (96%)	98% (5%)	3.1% (-4.5, 10.7)	
	Provider-school	13	73/74 (99%)	96% (14%)	1.2% (-6.5 9.0)	
How to use an RDT^‡^ ^(mean score, SD)^	Control	12	6.4 (3.1)	6.8 (3.2)	0	0.27
	Provider	14	7.9 (3.4)	7.6 (1.3)	0.9 (-0.9, 2.6)	
	Provider-school	13	9.1 (2.8)	8.2 (1.8)	1.4 (-0.3, 3.2)	
How to interpret an RDT result^§^	Control	12	28/54 (52%)	57% (38%)	0	0.92
	Provider	14	52/90 (58%)	61% (34%)	4.1% (-27.2, 35.5)	
	Provider-school	13	44/63 (70%)	63% (44%)	6.5% (-25.5, 38.4)	
First line treatment recommended by the Government	Control	12	42/68 (62%)	75% (40%)	0	0.18
	Provider	14	85/93 (91%)	86% (21%)	10.5% (-10.5, 31.6)	
	Provider-school	13	67/74 (91%)	96% (9%)	20.1% (-1.3, 41.5)	
ACT given if the malaria test is positive	Control	12	68/70 (97%)	84% (30%)	0	0.06
	Provider	14	104/106 (98%)	98% (5%)	13.9% (0.1, 27.6)	
	Provider-school	13	73/73 (100%)	99% (2%)	15.6% (1.6, 29.6)	
Antimalarial not given if the malaria test is negative	Control	12	56/71 (79%)	75% (32%)	0	0.60
	Provider	14	94/105 (89%)	83% (24%)	8.6% (-13.0, 30.3)	
	Provider-school	13	59/65 (91%)	85% (25%)	10.5% (-11.6, 32.5)	
**COMMUNITY KNOWLEDGE**						
Febrile patients should be tested for malaria^ǁ^	Control	12	61/85 (72%)	77% (25%)	0	0.85
	Provider	14	94/116 (81%)	75% (25%)	-2.1% (-22.4, 18.3)	
	Provider-school	14	97/115 (84%)	81% (31%)	3.5% (-17.2, 24.2)	
First line treatment recommended by the Government	Control	12	50/64 (78%)	79% (26%)	0	0.53
	Provider	13	70/81 (86%)	88% (18%)	8.4% (-9.1, 25.8)	
	Provider-school	14	112/126 (89%)	88% (20%)	8.5% (-8.6, 25.6)	
Were aware of a school or local community malaria event^#^	Control	12	64 /320 (20%)	20% (25%)	0	0.002
	Provider	14	30 /353 (9%)	9% (9%)	-10.7% (-29.3, 7.9)	
	Provider-school	14	110/288 (38%)	43% (31%)	22.5% (3.9, 41.1)	
Attended a school or local community malaria event^#^	Control	10	52/64 (81%)	66% (39%)	0	0.17
	Provider	10	25/30 (83%)	86% (30%)	21.3% (-5.1, 47.6)	
	Provider-school	12	89/108 (82%)	86% (17%)	21.2% (-4.0, 46.5)	

TABLE NOTES

* Number of providers – 75 in control, 110 in provider and 74 in provider-school. Number of households – 382 in control, 423 in provider, 413 in provider-school.

† Report that parasitological testing is recommended and that ACTs are for confirmed cases of malaria.

‡ Data are mean (SD): based on a score (out of 11) derived from correct identification of several steps taken in the use of an RDT. Steps include: Wear gloves; Write patient's name; Warm patient's finger; Clean patient's finger; Use lancet to prick finger; Dispose of lancet; Use loop to collect blood; Drop blood in well; Dispose of loop; Add buffer; Read results after 10–15 minutes. Sub-set of those who correctly identified that an RDT is used to diagnose malaria

^§^ Knows how to identify positive, negative, and invalid malaria RDT results

# May or may not be a REACT-initiated malaria event at school (some schools were used to distribute ITNs). Attended an event only asked of those who were aware of malaria activities in the schools or community in past year.

ǁ Among those who reported that they had heard about malaria diagnostic tests or RDTs

### Costs

Public facilities were supplied RDTs free of charge and although providers reported that they did not charge their patients for the RDTs some patients reported paying for them. Across all arms the median charge of RDTs in pharmacies and drug stores was 100 Naira (US$0.6), as reported by the provider. However, there was considerable variation in charges with patients commonly reporting higher costs (Table 5). The median cost of ACT was similar across all arms.

**Table 5:** Financial Costs of Intervention Design and Implementation, and costs of diagnosing and treating suspected malaria (USD 2011 prices).

	Control Arm	Provider Arm	Provider-school Arm
**Financial costs of intervention development and implementation**			
**Start-Up Costs**			
Engaging stakeholders	5, 055	5, 055	5, 055
Development of training materials	7, 128	28, 512	56, 516
**Implementation Costs**			
Demonstration on how to use RDTs	1, 384	-	-
Provider training workshop	-	8, 156	8,156
Support visits to providers*	-	950	847
Training workshop on school-based intervention	-	-	9,135
School malaria activities*	-	-	4,788
**TOTAL**	**13,567**	**42,672**	**84,496**
**Cost (median, range) of diagnosis and treatment of suspected malaria**			
**RDT cost to patients (provider reported)** ^**†**^	**(Naira)(USD)**	**(Naira)(USD)**	**(Naira)(USD)**
Public facility	0	0	0
Pharmacy	100 (50–250)0.6 (0.3–1.5)	100 (80–200)0.6 (0.48–1.2)	100 (100–650)0.6 (0.6–3.9)
Drug store	100 (50–200)0.6 (0.3–1.2)	100 (50–1100)0.6 (0.3–6.6)	100 (50–500)0.6 (0.3–3.0)
**RDT cost to patients (patient reported)**			
Public facility	0 (0–350)0 (0–2.1)	0 (0–200)0 (0–1.2)	0 (0–50)0 (0–0.3)
Pharmacy	100 (100–800)0.6 (0.6–4.8)	100 (100–150)0.6 (0.6–0.9)	150 (100–950)0.9 (0.6–5.7)
Drug store	150 (50–1200)0.9 (0.3–7.2)	150 (50–500)0.9 (0.3–3.0)	200 (50–1200)1.2 (0.3–7.2)
**ACT cost (patient reported)** ^**‡**^			
Public facility	50 (0–1500)0.3 (0–9.0)	0 (0–4000)0 (0–24.0)	0 (0–3800)0 (0–22.80)
Pharmacy	500 (130–1100)3.0 (0.8–6.6)	550 (150–4200)3.3 (0.9–25.2)	550 (50–1870)3.3 (0.3–11.22)
Drug store	400 (100–1790)2.4 (0.6–10.7)	400 (150–1200)2.4 (0.9–7.2)	450 (100–1550)2.7 (0.6–9.3)
Overall	420 (0–1790)2.5 (0–10.7)	450 (0–4200)2.7 (0–25.2)	500 (0–3800)3.0 (0–22.8)

TABLE NOTES

* The cost of the support visits and the school malaria events reflects the actual number of visits and events held.

† RDTs were supplied free of charge to facilities.

‡ The cost of the ACT medicine that the patient was prescribed or received.

The design and implementation cost of the interventions was $13,567 for RDTs with basic instruction (control) which is approximately one-third the cost of the provider intervention and one-sixth the cost of the provider-school intervention (Table 5). A full cost-effectiveness analysis was not considered necessary given the higher costs and minimal effect in the provider and provider-school intervention arms.

## Discussion

The interventions did not lead to a significant increase in the proportion of patients treated in accordance with malaria treatment guidelines. It is conceivable that this was driven by persistently low levels of testing across all arms. There were, however differences by type of facility with a higher proportion of patients tested in public facilities compared to private sector pharmacies and drug stores. The extent to which price could have contributed to the poor uptake of testing in private facilities requires further investigation. Despite most private providers reporting to have charged the recommended price of 100 Naira (US $0.60), there was considerable variation with some providers and patients reported to have paid up to ten times the recommended price. This suggests strategies to expand access to RDTs in the private sector may need to be accompanied by extensive activities to promote affordable testing.

One explanation for the lack of an effect may be that the interventions were not sufficiently different to yield observable differences, especially given the low proportion of patients tested. For instance, the instruction on how to use RDTs (control) covered some of the material from the provider training. Also, the interventions were evaluated in a trial to approximate the real-world setting, and this meant variation in the uptake of the intervention was not only permissible but also expected. For example, in the provider-school arm, uptake was not as anticipated with just under half of participating schools organising a malaria event. This demonstrates the reality of complex behaviour change interventions that rely on the goodwill of individuals to participate.

The performance of RDTs in ideal settings has been well established through a number of efficacy trials [31]. The aim of this study was not to replicate such ideal conditions but instead allowed for variability in health services behaviour and the subsequent effect on the implementation of our interventions. Contextual variability was especially evident in relation to drug supply. For instance, we found that the timing of the evaluation coincided with a period in which public facilities experienced major shortages of ACT and since the study did not provide any buffer stock, the effect of the interventions may have been curtailed by the availability of the recommended medicine. Data from the process evaluation also suggests that the effect of the interventions on the primary outcome may have been diluted by other interventions. We found a relatively large proportion of providers, even in the private-sector pharmacies and drug stores, reported they had received malaria training in the past two years, making it difficult to ascertain the effect of the latest training; although the pre- training tests showed there were still some gaps in their knowledge. In addition, responses to the household survey indicated that some participating schools had been used to distribute mosquito nets in the past year and this may have influenced household members when responding to questions about their awareness and attendance at a school malaria event.

Some aspects of the analysis are noteworthy. The number of facilities included in the study in each community (cluster) varied, resulting in considerable variation in cluster size in terms of the number of patients per cluster. Treatment according to guidelines was more common in larger clusters, with this association being more pronounced in the intervention arms. This resulted in the intervention effects having different directions in the individual- and cluster-level analyses, although in neither were they statistically significant. Stratifying on facility type–public or private–made the direction of the effects more consistent between the two types of analysis.

## Conclusions

The challenges of designing and implementing effective behaviour change interventions should not be under-estimated. Key policy questions remain and further research is needed to identify supporting interventions that could achieve universal diagnostic testing for malaria in this setting. Moreover, given the challenges we have outlined, it will be particularly important to consider whether it is cost-effective for the government to support the roll-out of RDTs in the private sector and we would recommend further investigation of this issue.

## Supporting Information

S1 CONSORT Checklist (PDF)

S1 Protocol (PDF)

## References

[1] WHO. World Health Organization (2010) Guidelines for the treatment of malaria, second edition World Health Organization, Geneva Available: http://www.who.int/malaria/publications/atoz/9789241547925/en/index.html. Accessed 18 June 2013.

[2] Cohen J, Fink G, Berg K, Aber F, Jordan M, Maloney K, et al (2012) Feasibility of distributing rapid diagnostic tests for malaria in the retail sector: evidence from an implementation study in Uganda. PloS one 7(11):e48296 10.1371/journal.pone.0048296 23152766 PMC3495947

[3] Bastiaens GJ, Bousema T, Leslie T (2014) Scale-up of malaria rapid diagnostic tests and artemisinin-based combination therapy: challenges and perspectives in sub-Saharan Africa. PLoS Med 11(1):e1001590 10.1371/journal.pmed.1001590 24465186 PMC3897367

[4] Williams HA, Causer L, Metta E, Malila A, O'Reilly T, Abdulla S, et al (2008) Dispensary level pilot implementation of rapid diagnostic tests: an evaluation of RDT acceptance and usage by providers and patients-Tanzania, 2005. Malaria J 7:239. 10.1186/1475-2875-7-239 PMC2613413 19019233

[5] Rowe AK, de Leon GF, Mihigo J, Santelli AC, Miller NP, Van-Dúnem P (2009) Quality of malaria case management at outpatient health facilities in Angola. Malaria J 8:275. 10.1186/1475-2875-8-275 PMC2795764 19954537

[6] Chandler CI, Whitty CJ, Ansah EK (2010) How can malaria rapid diagnostic tests achieve their potential? A qualitative study of a trial at health facilities in Ghana. Malaria J 9:95. 10.1186/1475-2875-9-95 PMC2859355 20398262

[7] Kyabayinze DJ, Asiimwe C, Nakanjako D, Nabakooza J, Counihan H, Tibenderana JK (2010) Use of RDTs to improve malaria diagnosis and fever case management at primary health care facilities in Uganda. Malaria J 9:200. 10.1186/1475-2875-9-200 PMC2914063 20624312

[8] Mbacham WF, Evehe MS, Netongo PM, Ateh IA, Mimche PN, Ajua A, et al (2010) Efficacy of amodiaquine, sulphadoxine-pyrimethamine and their combination for the treatment of uncomplicated Plasmodium falciparum malaria in children in Cameroon at the time of policy change to artemisinin-based combination therapy. Malaria J 9:34. 10.1186/1475-2875-9-34 PMC2831903 20105282

[9] Mangham LJ, Cundill B, Ezeoke O, Nwala E, Uzochukwu BS, Wiseman V, et al (2011) Treatment of uncomplicated malaria at public health facilities and medicine retailers in south-eastern Nigeria. Malaria J 10:155. 10.1186/1475-2875-10-155 PMC3120734 21651787

[10] Hamer DH, Ndhlovu M, Zurovac D, Fox M, Yeboah-Antwi K, Chanda P, et al Improved diagnostic testing and malaria treatment practices in Zambia. JAMA 297(20):2227–31. 17519412 10.1001/jama.297.20.2227 PMC2674546

[11] Ansah EK, Narh-Bana S, Epokor M, Akanpigbiam S, Quartey AA, Gyapong J, et al (2010) Rapid testing for malaria in settings where microscopy is available and peripheral clinics where only presumptive treatment is available: a randomised controlled trial in Ghana. BMJ 340:c930 10.1136/bmj.c930 20207689 PMC2833239

[12] Leslie T, Mikhail A, Mayan I, Cundill B, Anwar M, Bakhtash SH, et al (2014) Rapid diagnostic tests to improve treatment of malaria and other febrile illnesses: patient randomised effectiveness trial in primary care clinics in Afghanistan. BMJ 348:g3730 10.1136/bmj.g3730 24948695 PMC4064827

[13] Onwujekwe O, Uzochukwu B, Dike N, Uguru N, Nwobi E, Shu E (2009) Malaria treatment perceptions, practices and influences on provider behaviour: comparing hospitals and non-hospitals in south-east Nigeria. Malaria J 8:246. 10.1186/1475-2875-8-246 PMC2775747 19863803

[14] Uzochukwu BS, Onwujekwe E, Ezuma NN, Ezeoke OP, Ajuba MO, Sibeudu FT (2011) Improving rational treatment of malaria: perceptions and influence of RDTs on prescribing behaviour of health workers in southeast Nigeria. PloS One 6(1):e14627 10.1371/journal.pone.0014627 21297938 PMC3031496

[15] Federal Ministry of Health Nigeria. Federal Republic of Nigeria: National Antimalarial Treatment Policy Abuja, Nigeria; 2005 http://appswhoint/medicinedocs/documents/s18401en/s18401enpdf Accessed 18 August 2014.

[16] Onwujekwe O, Hanson K, Uzochukwu B, Ezeoke O, Eze S, Dike N (2010) Geographic inequities in provision and utilization of malaria treatment services in southeast Nigeria: diagnosis, providers and drugs. Health Policy 94(2):144–9. 10.1016/j.healthpol.2009.09.010 19836852

[17] Ezeoke OP, Ezumah NN, Chandler CC, Mangham-Jefferies LJ, Onwujekwe OE, Wiseman V et al (2012) Exploring health providers' and community perceptions and experiences with malaria tests in South-East Nigeria: a critical step towards appropriate treatment. Malaria J 11:368. 10.1186/1475-2875-11-368 PMC3507662 23130706

[18] Wiseman V, Ogochukwu E, Emmanuel N, Lindsay JM, Bonnie C, Eloka JE, et al (2012) A cost-effectiveness analysis of provider and community interventions to improve the treatment of uncomplicated malaria in Nigeria: study protocol for a randomized controlled trial. Trials 13:81 10.1186/1745-6215-13-81 22682276 PMC3517748

[19] Paul-Ebhohimhen VA, Poobalan A, van Teijlingen ER (2008) A systematic review of school-based sexual health interventions to prevent STI/HIV in sub-Saharan Africa. BMC Public Health 8:4 10.1186/1471-2458-8-4 18179703 PMC2248569

[20] Ayi I, Nonaka D, Adjovu JK, Hanafusa S, Jimba M, Bosompem KM, et al (2010) School-based participatory health education for malaria control in Ghana: engaging children as health messengers. Malaria J 9:98. 10.1186/1475-2875-9-98 PMC2865503 20398416

[21] Nonaka D, Kobayashi J, Jimba M, Vilaysouk B, Tsukamoto K, Kano S, et al (2008) Malaria education from school to community in Oudomxay province, Lao PDR. Parasitol Int 57(1):76–82. 17980652 10.1016/j.parint.2007.09.005

[22] Okabayashi H, Thongthien P, Singhasvanon P, Waikagul J, Looareesuwan S, Jimba M, et al (2006) Keys to success for a school-based malaria control program in primary schools in Thailand. Parasitol Int 55(2):121–6. 16406685 10.1016/j.parint.2005.11.056

[23] Shu EN, Onwujekwe EO, Lokili P, Okonkwo PO (2000) A health club for a community school in south-eastern Nigeria: influence on adult perception of onchocerciasis and compliance with community-based ivermectin therapy. Trop Med Int Health 5(3):222–6. 10747286 10.1046/j.1365-3156.2000.00531.x

[24] Shu EN, Okonkwo PO, Onwujekwe EO (1999) Health education to school children in Okpatu, Nigeria: impact on onchocerciasis-related knowledge. Public Health 113(5):215–8. 10557114

[25] Shu EN, Nwadike KI, Onwujekwe EO, Ugwu OC, Okonkwo PO (1999) Influence of health education on community participation in rapid assessment of onchocerciasis prior to distribution of ivermectin. E Afr Med J 76(6):320–3. 10750518

[26] Heeren T, D'Agostino R (1987) Robustness of the two independent samples t-test when applied to ordinal scaled data. Stat Med 6(1):79–90. 3576020 10.1002/sim.4780060110

[27] Donner A, Klar N (2000) Design and analysis of cluster randomization trials in health research London, Arnold.

[28] Bennett S, Parpia T, Hayes R, Cousens S. Methods for the analysis of incidence rates in cluster randomized trials. Int J Epidol 31(4):839–46. 10.1093/ije/31.4.839 12177032

[29] Rutstein AS, Johnson K (2004) The DHS wealth index. DHS comparative reports no. 6. Calverton: ORC Macro 2004. Available: http://wwwchildinfoorg/files/DHS_Wealth_Index_(DHS_Comparative_Reports)pdf Accessed 18 August 2014.

[30] Vyas S, Kumaranayake L (2006) Constructing socio-economic status indices: how to use principal components analysis. Health Policy Plann 21(6):459–68. 10.1093/heapol/czl029 17030551

[31] Abba K, Deeks JJ, Olliaro PL, Naing C-M, Jackson SM, Takwoingi Y, et al (2012) Rapid diagnostic tests for diagnosing malaria. Cochrane review. Available: http://www.cochrane.org/CD008122/INFECTN_rapid-diagnostic-tests-for-diagnosing-malaria Accessed 9 June 2015.

